# Medical Society of the County of Albany, Semi-Monthly Meeting, Jan. 11th and 24th, 1872

**Published:** 1872-02

**Authors:** James S. Bailey


BUFFALO

Medical and Surgical Journal.

VOL. XI. FEBRUARY, 1872. No. 7.

### Original Communications.

ART. I.—*Medical Society of the County of Albany. Semi-Monthly Meeting, January 11th, and 24th,* 1872. *Reported by* JAMES S. BAILEY, M. D.

DR. JOSEPH LEWI, President, in the chair.

DR. A. VAN DERVEER reported the following case of *Amputation of the Cervix Uteri*:

Mrs. A—, æt. 24, married 6 years, began to menstruate at 15, and soon after noticed appearances of the cervix or os externum between the external labia; menstruation always attended with great pain. Dr. V. first saw her January, 1871, and diagnosticated. "Longitudinal hypertrophy of the cervix." The fundus and body of the uterus was normal, the uterus measured five and one-fourth inches; the cervix measured three inches. The operation was performed March 23rd, 1871, by slitting up the cervix uteri, and with curved scissors removing from two to two and a half inches of cervix. The wound was closed with silver sutures. Union took place speedily. She menstruated April, May, June and July without pain. In August she conceived, and over-reaching in cleaning house, aborted Nov. 15th, 1871. The cervix now presented the same appearance as usual after parturition. Dr. VanDerveer also exhibited to the society a *polypns* extracted from the posterior nares also a diseased kidney that had undergone fatty degeneration.

DR. AMOS FOWLER reported the following case: Was called to see a lady in Oct., 1870, who was suffering with convulsions, the se were succeeded by paralysis on the right side of the body. After lying some days in a comatose condition she gradually recovered her senses, and in three or four weeks, sensation gradually returned. After this any unusual excitement precipitated convulsions. The urine was scanty, high-colored, and heavily laden with phosphates, constipation was also present. After a succession of various symptoms, in about a year after the attack, blisters appeared upon the face resembling erysipelas ; these disappeared, but attacked the limbs and arm of the left or well side. These parts also became livid. She became deaf in one ear and blind in one eye. Last summer she had a convulsion, bled freely at the nose and mouth, and the arm speedily regained its normal color and strength. Diagnosis : Apoplectic clot on the brain.

DR. J. S. BAILEY ascribed the purple color of the arm and leg to a lack of vitality, he also reported a case of *hemiplegia*, which on investigation proved to be hysteria.

The following named gentlemen were proposed for membership : Drs. F. C. Curtis, J. B. Stonehouse, Jr., and E. R. VanSlyke, all of which were referred to the comittiæ minora.

Dr. CHAS. DEVOL then remarked upon *Enteric obstructions*, and divided the subject into two distinct headings, viz : I.—Those cases which are due to accumulations of feculent matter or foreign substances in the intestines, either in the small intestines, or at the capul coli, and in the colon. II.—Those due to accumulation in the rectum so hard as to fill the pelvic cavity and make an enormous tumor in the perinæum. In the first class two illustrative cases were given, viz : Wm. S—, middle aged teamster, suffering for three days from what was supposed to be bilious colic, and when seen by Dr. D. had hiccough, cold clammy sweat and stercoracious vomiting; percussion and manipulation disclosed a small hard tumor in the region of the capulcoli, by placing the patient upon his hands and knees enemas were given, when a ball of potato skins were voided compact, two inches in diameter. Now all outward symptoms subsided, and the patient was restored to his accustomed health. 2d case.—A boy enormously corpulent, a gormandizer, eating everything within his reach, examination revealed the abdomen distended with hard feculent matter. The patient would seemingly fall down in spasms, and lay helpless for five or ten minutes, then get up and walk around as usual. When Dr. D. was called, his bowels were constipated, not having moved for seven days. Diagnosis: Enteric obstruction. By the use of enemas excrements of four weeks accumulations were voided which bore all the markings and corrugations of the small and large intestines and rectum.

This matter measured more in length than the boy; result, recovery. In the second class enemas are useless, digital operations alone succeed, cases of elderly people; women suffering in this way, also another class was mentioned, viz: scirrhus of the rectum or vagina, or both. Dr. D. remarked that feculent matter was discharged in a fluid state in such conditions, the fæces decomposing and becoming liquid above the point of obstruction. These cases were hopeless and incurable; the treatment must be expectant and palliative. The speaker attributed attacks of the majority of cases of dysentery to partial obstructions and retention of feculent matter. Dr. Thompson, Sabin, Boyce, Davis and others participated in the discussion of the subject, each mentioning interesting cases.

Dr. J. N. Northrop related a case of tumor in the right hyperchondrium, palpable to the touch, and very sensitive, which had been noticed for months, was accompanied with retching, fever, and frequent desire to defecate. Enemas and opiates were used with only partial relief. He then gave aloes gr. iv., of nux vomica gr. i., veratrum viride, m. 4, several times during the day, when on the fourth day large feculent masses, as large as the fist were discharged, much to the relief of the patient.

Dr. R. H. Sabin reported the following case of *Gastro Enteritis*. Male, æt. 53 years, carpenter, has been troubled with indigestion two years ago, and consequently had poor health, pains in the bowels and stomach, with vomiting. Oct. 22d he consulted Dr. S., when he discovered liquid in the abdomen. Blisters temporarily relieved the pain, but ascites increased and death took place. Nov. 25th a post mortem revealed complete destruction of the peritoneum, the liquid being shreddy. The mucous membrane of the stomach and bowels were highly inflamed, and the rectum completely obliterated.

Dr. James S. Bailey arose and said that as Dr. Milton M. Lamb, for fifteen years a member of the Society, had decided to remove to Lansingburgh to relieve the professional labors of his father-in-law, Dr. Davis, and as he had by his quiet and courteous deportment and estimable character, gained the respect and confidence of a large circle of acquaintances and retained their complete confidence during this period. The society owed him an expression of their good will. He therefore offered the following:

WHEREAS, Dr. Milton M. Lamb is about leaving this city for the purpose of residing and practicing his profession in the town of Lansingburgh, therefore

*Resolved*, That it is with regret that this society part with its honored associate and member, and hope that he may receive in his new abode, that courtesy and kind consideration to which his urbanity of manners and his professional conduct have ever entitled him; that Dr. Lamb carries with him, in leaving this city, the good wishes and respect of every member of the Albany County Medical Society, of which he has been a member for the last 15 years; that this society wish Dr. Lamb happiness in all his relations of life and continued success in his profession, which his professional attainments so richly deserve. Adopted.

DR. P. J. C. W. GOLDING reported the following case of *Catalepsy*: J. B—, æt. 40, Irish bar tender, Dec. 17th, 1871. After waiting on some customers, was taken with a fit, with sudden loss of sensation and volition, the body and limbs remaining in and retaining the position which they had when seized; the eyes were open and fixed, pulse natural, the color and respiration normal, no rigidity. By restoratives he regained his consciousness, began to bleed freely. He remained about four hours in the fit. The second day after the attack he was taken into the country, and has since enjoyed the best of health. Dr. G. then said that intenseness of thought, cessation of the catamenia, and terror have been ascribed as the causes of this malady. Watson asserts that catalepsy must depend on "congelation of the nervous fluid," and Hoffmann considers that freezing produces first catalepsy and then death. The Dr. ascribed the cause of this attack in the man Burns more to the "judgment of God in answer to a wicked and profane prayer," than to any form of terror. It appears that Burns was accused by his employer of appropriating to himself money belonging to the latter. In asserting his innocence Burns "sought the interposition of the Almighty, begging of God to strike him dead before morning if he had taken the money.

The employer went to obtain legal advice, and on his return found his attendant in the cataleptic state. Dr. G. was inclined to the opinion that this case partook more of the nature of a Divine judgment than terror superinduced by the contemplation of criminal proceedings in criminal courts of law.

Dr. SABIN then related a case which he considered catalepsy, but which was more like somnambulism than anything else.

Dr. D. V. O'Leary reported the following: July 23d was called to see B. R., æt. 21, suffering from compound fracture of the leg, the tibia protruding through the integuments. Tetanic convulsions set in on the 23d day of July. This gentleman was traveling with a circus, was thrown from a wagon when the fracture was produced. Dr. Hill of Canandaigua placed the limb in a fracture box. The convulsions lasted 14 days, the addominal and muscles of the neek were much contracted. Opium in large doses failed to produce sleep or to moderate the spasms. Hydrate Chloral in half-drachm doses was administered every four hours, when sleep was procured for three or four successive hours. The Chloral was continued for fourteen days, one day only was it omitted, as no medicine could be given by the mouth, then chloroform was administered by inhalation. Recovery eventually took place, and the fracture united with only a half inch shortening.

